# Parental feeding practices and child weight status in Mexican American families: a longitudinal analysis

**DOI:** 10.1186/s12966-015-0224-2

**Published:** 2015-05-20

**Authors:** Jeanne M. Tschann, Suzanna M. Martinez, Carlos Penilla, Steven E. Gregorich, Lauri A. Pasch, Cynthia L. de Groat, Elena Flores, Julianna Deardorff, Louise C. Greenspan, Nancy F. Butte

**Affiliations:** Department of Psychiatry, University of California at San Francisco, 94143-0848 San Francisco, CA USA; Department of Pediatrics, University of California at San Francisco, 94143-0503 San Francisco, CA USA; School of Public Health, University of California at Berkeley, 50 University Hall, 94720-7360 Berkeley, CA USA; Department of Medicine, University of California at San Francisco, 94143-0856 San Francisco, CA USA; Counseling Psychology Department, School of Education, University of San Francisco, 2130 Fulton Street, 94118 San Francisco, CA USA; Division of Community Health and Human Development, School of Public Health, University of California at Berkeley, 50 University Hall, 94720-7360 Berkeley, CA USA; Kaiser Permanente, 2200 O’Farrell Street, 94115 San Francisco, CA USA; Baylor College of Medicine, USDA/ARS Children’s Nutrition Research Center, Department of Pediatrics, 1100 Bates Street, 77030-2600 Houston, TX USA

**Keywords:** Feeding practices, Mexican Americans, Latinos, Child, Body mass index, Obesity, Parents, Fathers, Longitudinal

## Abstract

**Background:**

Parental feeding practices are thought to influence children’s weight status, through children’s eating behavior and nutritional intake. However, because most studies have been cross-sectional, the direction of influence is unclear. Moreover, although obesity rates are high among Latino children, few studies of parental feeding practices have focused on this population.

**Methods:**

This 2-year longitudinal study examined mutual influences over time between parental feeding practices and children’s weight status, in Mexican American families with children 18 years old at baseline. Mothers (*n* = 322) and fathers (*n* = 182) reported on their feeding practices at baseline, 1-year follow-up, and 2-year follow-up. Weight status, defined by waist-height ratio (WHtR) and body mass index (BMI), was ascertained at all assessments. Cross-lagged panel models were used to examine the mutual influences of parental feeding practices and child weight status over time, controlling for covariates.

**Results:**

Both mothers’ and fathers’ restriction of food predicted higher subsequent child weight status at Year 1, and for fathers this effect was also found at Year 2. Mothers’ and fathers’ pressure to eat predicted lower weight status among boys, but not girls, at Year 1. Child weight status also predicted some parental feeding practices: boys’ heavier weight predicted mothers’ less pressure to eat at Year 1, less use of food to control behavior at Year 2, and greater restriction at Year 2; and girls’ heavier weight at Year 1 predicted fathers’ less pressure to eat and less positive involvement in child eating at Year 2.

**Conclusions:**

This study provides longitudinal evidence that some parental feeding practices influence Mexican American children’s weight status, and that children’s weight status also influences some parental feeding practices. Feeding practices of both mothers and fathers were related to children’s weight status, underscoring the importance of including fathers in research on parental feeding practices and child obesity.

**Electronic supplementary material:**

The online version of this article (doi:10.1186/s12966-015-0224-2) contains supplementary material, which is available to authorized users.

The high prevalence of obesity among children is of great concern. Obese children are likely to be obese as adults; and obesity is a risk factor for type 2 diabetes, cardiovascular disease, and sleep apnea [1–4]. Mexican American children have an elevated prevalence of obesity, compared to non-Hispanic white children. Among children 6–11 years old, 22.4 % of Mexican American girls and 21.8 % of Mexican American boys were obese in 2009–2010, compared with 10.7 % of non-Hispanic white girls and 16.8 % of non-Hispanic white boys [5].

There is a critical need to identify modifiable risk factors for obesity among Mexican American children. One important influence on children’s weight, for which interventions could be developed, is parental feeding practices. Parental feeding practices are thought to influence children’s weight gain, through children’s eating behavior and nutritional intake. Controlling feeding practices, such as restriction of food, pressure to eat, and use of food to control behavior, may cause children to focus on external cues and impede their ability to self-regulate their food intake [6, 7]. In general, restricting foods appears to increase their desirability, while pressure to eat may reduce foods’ desirability [7]. Consistent with this conceptualization, most cross-sectional studies have reported that parents’ restriction of food is linked to children’s higher weight status [8–13], and pressure to eat is associated with children’s lower weight status [9–15]. In contrast, positive feeding practices, often conceptualized as a child-centered and including behaviors such as encouraging healthy eating and new foods, may allow children to develop self-regulation using their internal cues of hunger and satiety [16].

Longitudinal research can provide guidance for obesity prevention interventions. If parental feeding practices influence child weight status, such information could be incorporated into obesity prevention interventions; but if parental feeding practices are largely a response to child weight, then interventions could focus more on addressing parents’ concerns about their children’s weight. However, because most studies have been cross-sectional, the direction of influence between parental feeding practices and child weight status is unclear [12]. Moreover, the few existing longitudinal studies have reported inconsistent findings. In young children, two studies have reported that mothers’ pressure to eat predicted lower weight status [17, 18], one study found that restriction of food predicted lower weight status in contrast to cross-sectional studies [17], and one found that use of food as a reward predicted higher weight status [19]. Other longitudinal studies have found effects of parental feeding practices only among certain subgroups, such as children of overweight mothers [20, 21], younger children (ages 5–6) but not older children (ages 10–12) [22], or among boys but not girls [23]. Several longitudinal studies have found no effects of parental feeding practices on children’s subsequent weight [24–27].

Parents may also modify their feeding practices in response to children’s weight status, and this possibility has been examined in three longitudinal studies. Rhee and colleagues [23] found that for girls (but not boys) who had greater weight gain, mothers subsequently used more controlling feeding. Webber and colleagues [27] found that higher baseline child weight predicted increased maternal monitoring and reduced pressure to eat over a 3-year period. Finally, Jansen and colleagues [18] reported that higher baseline child weight predicted greater maternal restriction and less pressure to eat 2 years later.

Relatively few studies of parental feeding practices and children’s weight have focused specifically on Latino families [10, 14, 28–32]. Despite the fact that Latinos are the largest ethnic minority group in the U.S., and 63 % of Latinos are Mexican Americans [33], no previous longitudinal study of parental feeding practices and child weight status has focused on Mexican Americans or any Latino group.

The major purpose of this study was to examine the mutual influences between parental feeding practices and child weight over a 2-year period, in Mexican American families with children 8–10 years old. Both mothers and fathers participated in the research. Most previous cross-sectional studies of parental feeding practices have focused only on maternal feeding practices. To date, no longitudinal research has reported on whether fathers’ feeding practices influence children’s weight status, although evidence is beginning to emerge that fathers’ feeding practices are also associated with their children’s weight [34–39].

The current study examined four types of parental feeding practices: restriction of amount of food, pressure to eat, use of food to control behavior, and positive involvement in child eating. There is evidence that Latino parents use all of these feeding practices [10, 40]. Restriction of food and pressure to eat have been examined in numerous studies [12]. Use of food to control behavior has been studied less often, but a recent longitudinal study reported that using food as a reward – one aspect of using food to control behavior—predicted children’s weight gain one year later [19]. Finally, positive involvement in child eating was conceptualized as a child-centered feeding practice, encompassing monitoring of high-calorie foods, encouraging healthy eating and new foods, and providing small servings [10]. Among Mexican American children, greater maternal positive involvement in child eating has been linked to children’s lower weight status [10].

In this study, we examined whether parental feeding practices and child weight status influenced each other at three points over a 2-year period, in Mexican American families with children who were 8–10 years old at baseline. We hypothesized that parental feeding practices would predict child weight status: specifically, that restriction of food and using food to control behavior would predict increased child weight status, and that pressure to eat and positive involvement in child eating would predict lower child weight status. We also examined whether child weight status would predict parental feeding practices; specifically, whether greater child weight status would predict more restriction of food, less pressure to eat, and less use of food as a reward. We examined these relationships for both mothers’ and fathers’ feeding practices. We also assessed these relationships separately by child gender, because previous research suggests that parental feeding practices may influence boys and girls differently [23].

## Methods

### Participants

Parents of 322 Mexican American children ages 8–10 were enrolled in the research. Eligibility criteria for participation included: a mother of Mexican descent (Mexican or U.S. born), and a child between 8 and 10 years old, who had no major illnesses. Families were eligible whether or not fathers participated, but every effort was made to recruit fathers. If the father did not reside in the same household as the mother and child, the primary father figure (biological father living apart or residential parental figure) was recruited to participate. Of the 322 families participating in the study, 57 % (*n* = 182) of fathers participated.

### Procedure

We recruited families to participate in a 24-month longitudinal cohort study to understand parental influences on obesity in Mexican American children. Parents were members of Kaiser Permanente Northern California, an integrated health delivery system, between 2007 and 2009. Kaiser Permanente is one of the largest health care providers in California, with membership occurring through employer-provided insurance coverage, individual enrollment, or state-funded (Medi-Cal) programs. A computer program was used to select potential participants from a Kaiser Permanente membership list. Selection criteria were members with a Spanish surname and a child in the eligible age range. These parents were sent letters introducing the research, were telephoned by research assistants, were screened for eligibility, and if eligible, were invited to participate in the study. 37 % of eligible families participated in the research.

If the mother or both parents agreed to participate, a baseline assessment home visit was scheduled. At home visits, bilingual research assistants first obtained written parental informed consent and verbal child assent. Families were assessed at baseline (BL), 1-year follow-up (Yr1), and 2-year follow-up (Yr2). All study materials were developed in both Spanish and English, and interviews were conducted in the language of participants’ choice. Most parents chose to be interviewed in Spanish (71 % of mothers, 69 % of fathers). Research assistants interviewed family members individually in their homes, and recorded responses to the questionnaires in laptop computers. Research assistants also measured family members’ height, weight, and waist circumference. The in-home interview and assessment lasted about 5 hours per time point. The study was approved by the university and Kaiser Permanente Northern California institutional review boards.

### Measures

#### Parental feeding practices

At each assessment, parents completed the 55-item Parental Feeding Practices (PFP) Questionnaire [10] about the study child. The PFP was developed for use with Latino parents, and has good validity and reliability [10]. It includes items based on focus group discussions, as well as items adapted from previous measures, and contains four subscales: restriction of amount of food (12 items, e.g*.*, “How often do you tell your child he/she has eaten enough?”; α _mothers_ = 0.77, α _fathers_ = 0.70), pressure to eat (10 items, *e.g.*, “How often do you tell your child to eat everything on the plate?”; α _mothers_ = 0.86, α _fathers_ = 0.84), use of food to control behavior (9 items, *e.g.*, “How often do you give your child something to eat or drink to make him/her happy, even if you think he/she isn’t hungry?”; α _mothers_ = 0.78, α _fathers_ = 0.75), and positive involvement in child eating (24 items, *e.g.*, “How often do you find out how much your child ate during the day?”; α _mothers_ = 0.88, α _fathers_ = 0.91). All questions were worded in terms of frequency of behavior, and response options ranged from never (=1) to always (=5). For each subscale, mean scores were calculated; higher scores represented more use of that feeding practice. In previous research, mothers’ and fathers’ feeding practices scores were modestly to moderately correlated (rs = 0.19 - 0.46) [10]. In addition, most feeding practices subscales were related to children’s weight status for both parents (rs = 0.18–0.35). Exceptions were mothers’ use of food to control behavior and fathers’ positive involvement in child eating [10].

#### Children’s weight status: waist-height ratio (WHtR) and body mass index (BMI)

At each assessment, child height, weight and waist circumference were obtained using standard procedures; and in duplicate while the participants were wearing light indoor clothing and no shoes [41, 42]. Waist-height ratio (WHtR) was used as a measure of the distribution of central adiposity. This sensitive and specific marker of upper body fat is a good predictor of cardiovascular disease risk factors in children [43, 44]. WHtR was obtained by dividing the child’s waist circumference by their height. As a clinical measure, WHtR should be less than 0.5, reflecting the standard that an individual’s waist circumference should be less than half their height [45]. Children’s body mass index (BMI) was also calculated (weight[kg]/height[m]^2^). Raw BMI scores were used in analyses, because these allow for variability in extreme scores to be more accurately assessed over time, compared to BMI z-scores [46, 47].

#### Covariates: parent and child characteristics

We included several parental characteristics as possible covariates: family-level socioeconomic status (SES), acculturation, and parental BMI at BL. Family-level SES was a standardized score based on each parent’s years of education and occupational status. Occupational status could range from unskilled (=1) to major professional (=9) [48]. Acculturation was assessed using the Spanish Language Use and English Language Use subscales of the Bidimensional Acculturation Scale for Hispanics [49]. An example item is “How often do you speak English with your friends?” Items are scored from never (=1) to always (=5), and have good reliabilities (α for mothers and fathers = 0.88–0.94). Parents’ BMI was calculated (weight[kg]/height[m]^2^).

We also included child gender, age, and pubertal status at BL as potential covariates. Pubertal status has been associated with obesity in previous studies [50]. We used the 5-item Pubertal Development Scale [51], which was completed by mothers at BL. This measure, with versions for males and females, asks about physical development on characteristics associated with physical maturation, with response options ranging from *no* (=1) to *yes, a lot* (=3). Separate mean scores were calculated by gender.

### Statistical analyses

**Figure 1:**
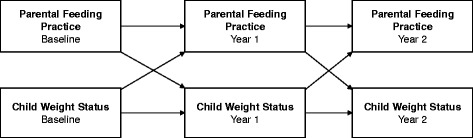
General cross-lagged panel model, showing mutual influences of parental feeding practices and child weight status across three time points

Pearson correlations were used to examine the relationships between covariates and child weight status (WHtR and BMI) at BL. Covariates that were significantly related to child weight status were included in multivariate analyses. We fit cross-lagged panel models to estimate the effects of parental feeding practices and child weight status on one another over time. Cross-lagged panel models are widely used with longitudinal data to examine the direction of influence between two variables that are measured repeatedly over time. Cross-lagged models provide estimates of regression coefficients between each variable measured at one wave and the other variable at the next wave. In the current study, there were three time points (BL, Yr1, Yr2), and the two variables measured at each time were parental feeding practices and child weight status (general model shown in Fig 1). Each cross-lagged model controlled for covariates at BL. Separate models were estimated for mothers’ and fathers’ four feeding practices, and for WHtR and BMI (16 models). Satorra-Bentler scaled Chi-square test statistics assessed goodness-of-fit of each model, and approximate model fit was examined using the recommendations of Hu and Bentler [52]; *i.e.*, root mean square error of approximation [RMSEA] ≤ 0.06, and standardized root mean square residual [SRMR] ≤ 0.08. Modeling was performed using Mplus 7, with full information maximum likelihood to accommodate missing values [53].

We also tested whether child gender modified the cross-lagged effects between each parental feeding practice and child weight status variable. This was accomplished by comparing the fit of a model that allowed cross-lagged effects to freely vary across child gender, to a model that constrained corresponding cross-lagged effects to be equal across child gender. A significant *χ*^2^ difference test would indicate significantly improved fit for the freely-estimated model – an omnibus test of the moderating effect of child gender. For freely-estimated models that showed significantly improved fit, we fit cross-lagged panel models separately by child gender.

A total of 246 mothers had complete data at all three time points, 44 were missing data at one time point, and 32 were missing data at two time points. A total of 98 fathers had complete data at all three time points, 67 were missing data at one time point, and 17 were missing data at two time points. All 322 mothers were included in the analyses of mothers’ feeding practices and all 182 fathers were included in the analyses of fathers’ feeding practices.

## Results

### Participant characteristics

Demographic characteristics of mothers, fathers and children are shown in Table 1. Additional demographic information included the following: most participating fathers were biological fathers living with the mothers (90 %); the remainder were stepfathers (7 %), or biological fathers living apart from the mothers (3 %). Most parents were born in Mexico (78 % mothers; 74 % fathers), while most participating children (95 %) had been born in the U.S. By design, all mothers were of Mexican heritage. Most fathers were also of Mexican heritage (83 %); the rest were other Latino heritage (9 %), or other/mixed ethnicities (8 %). Most parents were employed (75 % of mothers, 93 % of fathers). Parents’ average occupational status was skilled worker (=3). Descriptive statistics for parental feeding practices are shown in Table 1.

**Table 1:** Demographic characteristics and parental feeding practices in Mexican American families at baseline

	Mean (SD) or %		
	Mother	Father	Child
Variable (range)	(*n* = 322)	(*n* = 182)	(*n* = 322)
Parent characteristics			
Education (0–19 years)	10.77 (3.69)	11.02 (3.67)	
Occupational status (1–9)	3.25 (2.09)	3.53 (1.84)	
Acculturation (1–5)			
Spanish language	4.23 (1.10)	4.01 (1.10)	
English language	2.64 (1.27)	2.94 (1.11)	
BMI (18–72)	30.29 (6.69)	29.81 (4.33)	
Child characteristics			
Gender (% female)			53 %
Age (8–10 years)			9.29 (0.92)
Pubertal status (1–3)			1.10 (0.32)
Waist-height ratio (WHtR) (0.37–0.79)			0.50 (0.08)
BMI(14–48)			20.35 (4.75)
Parental feeding practices (1–5)			
Restriction of amount of food	2.28 (0.44)	2.29 (0.47)	
Pressure to eat	2.30 (0.86)	2.43 (0.85)	
Use of food to control behavior	1.50 (0.44)	1.62 (0.50)	
Positive involvement in meals	3.35 (0.69)	3.11 (0.70)	

Most parents were overweight (BMI ≥ 25 and <30; 33 % of mothers, 47 % of fathers) or obese (BMI ≥30; 48 % of mothers, 45 % of fathers). Based on age- and gender-specific BMI percentiles [54], 20 % of the children were overweight (≥85^th^ %ile, <95^th^ %ile) and 31 % were obese (≥95^th^ %ile) at BL (baseline). 42 percent of children had a WHtR > 0.50.

### Correlations between study variables

Correlations between covariates and BL child weight status are shown in Table 2. Based on significant correlations with child WHtR or BMI, covariates included in subsequent multivariate analyses were SES, Spanish language acculturation, parental BMI, child age, and child pubertal status. As shown in Table 3, most parental feeding practices were significantly related to child WHtR and/or BMI at BL, with the exception of positive involvement in child meals. Child WHtR and BMI were significantly related at BL, Yr1, and Yr2 (rs = 0.58, 0.84, 0.53, ps < 0.001, respectively).

**Table 2:** Correlations between demographics and child weight status in Mexican American families at baseline

Demographics	Child WHtR^1^	Child BMI
Family SES	−0.12*	−0.12*
Mother acculturation (Spanish)	0.14*	0.08
Mother acculturation (English)	−0.08	−0.03
Father acculturation (Spanish)	0.10	0.18*
Father acculturation (English)	−0.07	0.12
Mother BMI	0.21***	0.36***
Father BMI	0.30***	0.23***
Child gender	−0.01	0.00
Child age	0.14*	0.28***
Child pubertal status	0.22***	0.33***

*p < 0.05; **p < 0.01; ***p < 0.001

1. *WHtR* waist-height ratio

**Table 3:** Correlations between parental feeding practices and child weight status in Mexican American families at baseline

	Child waist-height ratio		Child BMI	
	Girls	Boys	Girls	Boys
Mothers’ feeding practices (*n* = 322)				
Restriction	0.38***	0.24**	0.51***	0.45***
Pressure	−0.18*	−0.27***	−0.34***	−40***
Use food to control	−0.09	−0.15	−0.24**	−0.22**
Positive involvement	−0.04	−0.11	−0.03	−0.05
Fathers’ feeding practices (*n* = 182)				
Restriction	0.37***	0.13	0.39***	0.47***
Pressure	−0.23*	−0.29**	−0.19	−43***
Use food to control	−0.11	−0.24*	−0.13	−0.25*
Positive involvement	−0.03	−0.09	0.05	0.01

*p < 0.05; **p < 0.01; ***p < 0.001

### Longitudinal relationships between parental feeding practices and child WHtR

Fig 1 illustrates the general model used to assess the relationships between parental feeding practices and child weight status over time, using cross-lagged panel models. For child WHtR, tests for interactions found that child gender significantly modified the cross-lagged effects between feeding practices and child WHtR in most models, including mothers’ restriction, pressure to eat, and use of food to control behavior; and fathers’ restriction, pressure to eat, and positive involvement in child eating. For these models, comparisons between freely estimated models and constrained models all showed that the freely estimated models had significantly improved fit (Table 4). All freely estimated models met the fit criteria (see Additional file 1). Therefore, cross-lagged panel model results for these models are reported separately by child gender. The models without significant interactions involving child gender – mothers’ positive involvement in child eating and fathers’ use of food to control behavior – also had no significant cross-lagged effects between feeding practices and child WHtR (data not shown).

**Table 4:** Comparisons of models with cross-lagged effects freely estimated across child gender and models with constrained cross-lagged effects, for waist-height ratio

Model	*S-Bχ* ^2^	df	*p*	∆*S-Bχ* ^2^	∆df	∆*p*
Mothers						
Restriction _Free_	39.56	36	0.31			
Restriction _Constrained_	92.43	49	<0.001	54.57	13	<0.001
Pressure _Free_	20.89	36	0.98			
Pressure _Constrained_	102.103	49	<0.001	67.20	13	<0.001
Control _Free_	24.21	36	0.93			
Control _Constrained_	82.31	49	<0.01	59.40	13	<0.001
Fathers						
Restriction _Free_	47.06	36	0.10			
Restriction _Constrained_	82.90	49	<0.01	36.06	13	<0.001
Pressure _Free_	38.85	36	0.34			
Pressure _Constrained_	97.07	49	<0.001	46.44	13	<0.001
Involvement _Free_	42.65	36	0.21			
Involvement_Constrained_	113.24	49	<0.001	58.64	13	<0.001

Note. *S-Bχ* ^2^: Satorra-Bentler *χ* ^2^ for ∆*S-Bχ* ^2^: Satorra-Bentler difference *χ* ^2^

∆df: difference in degrees of freedom between nested models

∆*p*: *p*-value for ∆*S-Bχ* ^2^

Cross-lagged panel model results for girls’ and boys’ WHtR, showing standardized regression coefficients, are summarized in Tables 5 and 6. (See Additional file 1 for figures.) All models included parental BMI, SES, Spanish language acculturation, and child pubertal status as covariates (covariates not shown in tables).

**Table 5:** Cross-lagged panel models: parental feeding practices predicting girls’ and boys’ weight-height ratio (WHtR)

	Mothers’ feeding practices		Fathers’ feeding practices	
Cross-lagged effects	Girls’ WHtR	Boys’ WHtR	Girls’ WHtR	Boys’ WHtR
	*β*	*β*	*β*	*β*
Baseline feeding practices → Year 1 child WHtR				
Restriction	0.23**	0.22**	0.21**	0.40***
Pressure	−0.12	−0.23**	−0.07	−0.29**
Use food to control	−0.10	−0.11	na	na
Positive involvement	na	na	0.10	0.08
Year 1 feeding practices → Year 2 child WHtR				
Restriction	−0.02	0.02	−0.11*	0.18*
Pressure	−0.01	−0.05	−0.04	−0.03
Use food to control	−0.03	0.02	na	na
Positive involvement	na	na	−0.07	0.15*

*p < 0.05; **p < 0.01; ***p < 0.001

na: not applicable because the interaction between child gender and feeding practice was not significant

**Table 6:** Cross-lagged panel models: girls’ and boys’ weight-height ratio (WHtR) predicting parental feeding practices

	Mothers’ feeding practices		Fathers’ feeding practices	
Cross-lagged effects	Girls’ WHtR	Boys’ WHtR	Girls’ WHtR	Boys’ WHtR
	*β*	*β*	*β*	*β*
Baseline child WHtR → Year 1 feeding practices				
Restriction	0.06	−0.00	0.07	−0.02
Pressure	−0.04	−0.20**	−0.02	0.01
Use food to control	−0.04	−0.03	na	na
Positive involvement	na	na	−0.04	-0.03
Year 1 child WHtR → Year 2 feeding practices				
Restriction	0.00	0.19*	0.07	0.03
Pressure	−0.10	−0.06	−0.20*	−0.07
Use food to control	−0.05	−0.31***	na	na
Positive involvement	na	na	−0.23**	0.09

*p < 0.05; **p < 0.01; ***p < 0.001

na: not applicable because the interaction between child gender and child WHtR was not significant

### Parental feeding practices predicting child WHtR

#### Mothers’ feeding practices

Mothers’ restriction at BL predicted girls’ higher WHtR at Yr1 (β = 0.23; Table 5). Mothers’ restriction at BL predicted boys’ higher WHtR at Yr1 (β = 0.22). Mothers’ pressure to eat at BL predicted boys’ lower WHtR at Yr1 (β = -0.23).

#### Fathers’ feeding practices

Fathers’ restriction at BL predicted girls’ higher WHtR at Yr1 (β = 0.21), but fathers’ restriction at Yr1 predicted girls’ lower WHtR at Yr2 (β = -0.11). Fathers’ restriction at BL and Yr1 predicted boys’ higher WHtR at subsequent years (βs = 0.40, 0.18, respectively). Fathers’ pressure to eat at BL predicted boys’ lower WHtR at Yr1 (β = -0.29). Fathers’ positive involvement in boys’ eating at Yr1 predicted boys’ higher WHtR at Yr 2 (β = 0.15).

### Child WHtR predicting parental feeding practices

#### Mothers’ feeding practices

Girls’ WHtR did not significantly predict mothers’ feeding practices. Boys’ higher WHtR at Yr1 predicted mothers’ greater restriction at Year 2 (β = 0.19; Table 6). Boys’ lower WHtR at BL predicted mothers’ less pressure to eat at Yr1 (β = -0.20). Boys’ lower WHtR at Yr1 predicted mothers’ less use of food to control behavior at Yr2 (β = -0.31).

#### Fathers’ feeding practices

Girls’ higher WHtR at Yr1 predicted fathers’ less pressure to eat at Yr2 (β = -0.20), as well as fathers’ less positive involvement in girls’ eating at Yr2 (β = -0.23). Boys’ WHtR did not significantly predict fathers’ feeding practices.

#### Parental feeding practices and child BMI

For child BMI, tests for interactions showed that child gender did not significantly modify the cross-lagged effects between feeding practices and child BMI in any model. Therefore, the constrained models for data pooled across child gender are reported. All models included parental BMI, SES, Spanish language acculturation, child pubertal status, and child age as covariates.

Models for mothers’ restriction, mothers’ use of food to control behavior, and fathers’ pressure revealed significant cross-lagged effects between feeding practices and child BMI, and had adequate fit. (See Additional file 1 for figures and fit statistics.) Parental feeding practices did not significantly predict child BMI in any of these models. However, child BMI predicted several parental feeding practices. Child greater BMI at BL predicted mothers’ greater restriction at Yr1 (β = 0.18). Child greater BMI at Yr1 predicted mothers’ less use of food to control behavior (β = - 0.14) and fathers’ less pressure to eat (β = - 0.21) at Yr2.

## Discussion

This research addresses the urgent need to identify modifiable risk factors for childhood obesity among Mexican American children. This longitudinal family-based study included both mothers and fathers in the research, examined the mutual influences of parental feeding practices and children’s weight status over time, and utilized the Parental Feeding Practices Questionnaire [10], which was validated for use with this population. Hypotheses were partially supported. Both mothers’ and fathers’ feeding practices, particularly restriction of amount of food and pressure to eat, predicted children’s subsequent weight status. However, parents’ positive involvement in child feeding and use of food to control child behavior had minimal or no influence on children’s subsequent weight status. Child weight status also predicted several parental feeding practices, with gender-specific findings: mothers altered some feeding practices in response to their sons’ weight status, and fathers altered some feeding practices in response to their daughters’ weight status.

A consistent pattern of findings was that parents’ use of food restriction predicted subsequent higher weight status in both girls and boys at Year 1. For fathers, this effect was also seen at Year 2, suggesting that fathers’ restriction in particular may have a continuing effect on child weight status. These findings are consistent with previous cross-sectional research [8–13], although most previous longitudinal studies found this link only among certain subgroups [20–22], or reported no significant effects [19, 24, 26, 27].

Parents’ use of pressure to eat predicted lower weight status at Year 1, among boys but not girls. Previous cross-sectional studies [9–15] and two longitudinal studies [17, 18] have reported that pressure to eat was related to children’s lower weight status, although several other longitudinal studies were unable to confirm this link [24, 26, 27]. However, none of the previous studies reported the effects of parental feeding practices on boys and girls separately, as we did in the present study.

Our findings regarding parental restriction and pressure to eat are longitudinal evidence that parents’ controlling feeding practices may have unintended influences on child weight status in Mexican American families. When parents attempt to restrict their children’s dietary intake, children subsequently tend to weigh more, and when parents urge their children to increase their food intake, boys tend to subsequently weigh less over time. Consistent with current theorizing [7], controlling feeding practices appear to increase Mexican American children’s reliance on external cues when eating.

There are several possible explanations for this study’s findings that parental restriction and pressure to eat influenced children’s weight status, in contrast to the nonsignificant findings of some previous longitudinal studies. First, we used waist-height ratio (WHtR), a measure of central adiposity, as well as body mass index (BMI) to assess children’s weight status, rather than the commonly used BMI z-scores [e.g., 19–20; 22–24]. Most of this study’s significant findings regarding parental influences on child weight status were those using WHtR. Because nearly a third of the study children were obese at baseline (*i.e.*, above the 95 % percentile of BMI scores), the WHtR measure may have yielded significant results due to its sensitivity to extreme scores [45]. Second, our sample of 322 mothers and 182 fathers was larger than those of most previous longitudinal studies; other larger studies also reported some significant findings [19, 21–23]. Third, we used an elaborated, culturally-based measure of parental feeding practices, developed and validated for use with this population [10]. Finally, some of our results were due to the inclusion of fathers and separate analyses by child gender. Overall, our findings suggest that some feeding practices likely do predict child weight status over time. More longitudinal research is needed in this area, particularly research using sensitive measures and large samples that include both mothers and fathers.

It is worth noting the particular effect of fathers’ feeding practices on boys’ weight status. When fathers engaged in more restriction, used less pressure to eat, and were more positively involved in their sons’ eating, their sons tended to have a higher weigh status a year later. These findings are intriguing, given that little is known about the feeding practices utilized by fathers, regardless of ethnicity [37]. Our results suggest that Mexican American fathers are involved in their children’s eating, and that future research including both parents in Mexican American families could illuminate the ways in which fathers and mothers interact with their sons and daughters regarding dietary intake. Moreover, future obesity interventions could be designed to include fathers as well as mothers as participants.

This is one of few longitudinal studies to examine the effects of child weight status on parental feeding practices. Parents appeared to alter some feeding practices in response to their children’s weight, in gender-specific ways. In particular, boys’ weight status predicted maternal feeding practices, while girls’ weight predicted fathers’ feeding practices. Mothers of boys with higher weight status subsequently used less pressure to eat at Year 1, more restriction of food at Year 2, and less use of food to control their sons’ behavior at Year 2. Fathers of girls with higher weight status at Year 1 subsequently engaged in less pressure to eat and were less positively involved in their daughters’ eating at Year 2. Our findings appear to be consistent with some previous longitudinal research with mothers and children, although those previous studies did not report results separately by child gender [18, 27]. However, our results contrast with those of Rhee and colleagues [23], who found that mothers of girls (but not boys) who gained more weight subsequently used more controlling feeding practices. Overall, our findings hint at the possibility that mothers and fathers have distinct parental roles regarding feeding practices, a topic that is beginning to receive some attention [37, 55, 56]. These cross-gender findings also underscore the importance of examining parental feeding practices separately by parents’ and children’s gender.

Our finding that children’s weight status predicts some parental feeding practices also suggests that parents who alter their feeding practices in response to their child’s weight may be experiencing concerns about their child’s weight. This notion is consistent with several cross-sectional studies reporting that maternal concerns about child weight are related to parental feeding practices (e.g., [27, 57, 58]). Parents may welcome interventions that could address these concerns and provide guidance for utilizing constructive feeding practices, such as recognizing children’s hunger and satiety cues while setting appropriate limits [59, 60].

This longitudinal study sheds light on the question of whether parental feeding practices influence children’s subsequent weight, or whether children’s weight influences parents’ subsequent feeding practices. We found some evidence for both directions of influence. Because this was not a randomized controlled trial, causal inferences cannot be drawn. Moreover, the sample was a convenience sample drawn from a large health care provider. However, a strength of this study was the fact that we assessed participants at three points in time, allowing for the mutual influences of parental feeding practices and child weight to be examined. Another limitation of this research is that results cannot be generalized beyond Mexican American families with mostly immigrant parents. The generalizability of the findings is also limited because only about one-third of eligible families participated in the research, possibly partially because of the considerable time commitment required. It would be of interest to investigate whether our findings also apply to other Latino subgroups, such as those who are more acculturated, as well as other cultural, ethnic, or economic groups; and whether the same findings would apply if research participation was less time-consuming. Moreover, because the PFP Questionnaire is relatively new, further investigation of its reliability and validity is needed. Finally, we studied only children ages 8–10 at baseline, and followed them for 2 years to ages 10–12. We speculate that the influence of parental feeding practices may be stronger at younger ages, as hinted by the findings of some previous research [17, 19, 22]. A valuable contribution to the literature would be to assess the ages at which the influence of parental feeding practices on child weight begins to diminish, as well as when parental responses to child weight begin to occur. Such information could be of use in designing future obesity prevention interventions.

## Conclusions

This study provides longitudinal evidence that parental feeding practices influence children’s weight status in Mexican American families, and that children’s weight status also influences parental feeding practices. Both mothers’ and fathers’ feeding practices appear to influence children’s weight status, underscoring the importance of including fathers in research on parental feeding practices and child obesity. Our findings suggest that both mothers and fathers should be included in obesity prevention interventions focusing on parental feeding practices in Latino populations. Finally, this longitudinal research adds to accumulating evidence regarding the undesirable effects of controlling feeding practices. Obesity prevention interventions may benefit from educating parents to avoid using controlling feeding practices – such as restriction of food and pressure to eat – from an early age, regardless of children’s weight. Toward this aim, interventions could address parents’ concerns about their children’s weight, by helping them to understand and be responsive to children’s hunger and satiety cues [60]. Interventions should also focus on healthy behaviors for the entire family, such as improved diet and physical activity, which help prevent childhood obesity [60, 61].

## Additional file

Additional file 1: Figures S2–14. Cross-agged panel models, showing mutual influences of parental feeding practices and child weight status across 3 time points.

## Abbreviations

BL: Baseline
Yr1: 1-year follow-up
Yr2: 2-year follow-up
WHtR: Waist-height ratio
BMI: Body mass index
PFP: Parental feeding practices questionnaire
