# Supplementary material for: Parental feeding practices and child weight status in Mexican American families: a longitudinal analysis
Source: Int J Behav Nutr Phys Act. 2015 May 20;12:66. doi: 10.1186/s12966-015-0224-2 (PMC4453102; doi:10.1186/s12966-015-0224-2)
Supplement: Additional file 1: Figures S2–14. — Cross-agged panel models, showing mutual influences of parental feeding practices and child weight status across 3 time points. [file 12966_2015_224_MOESM1_ESM.pptx]

## Slide 1
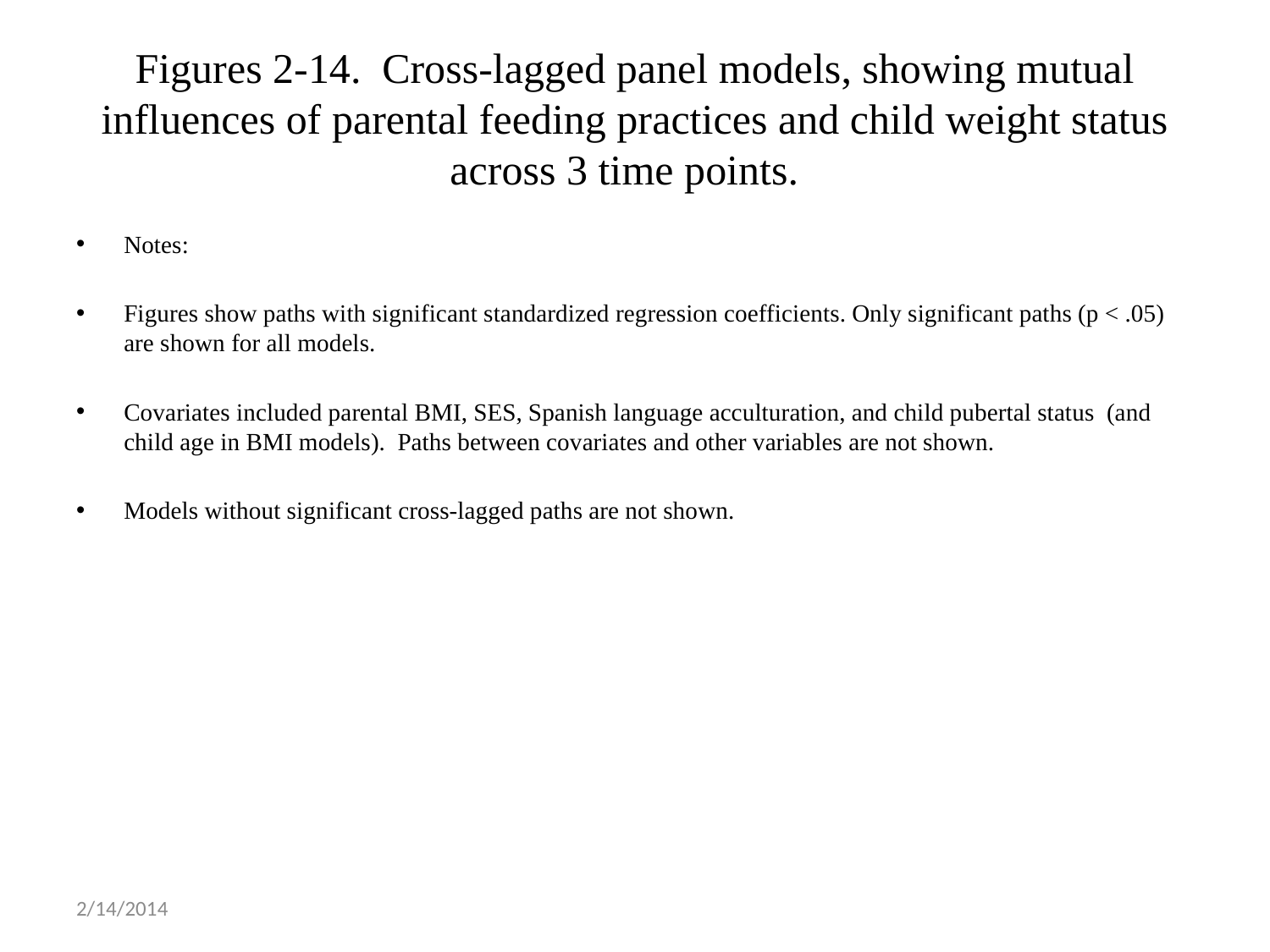

# Figures 2-14. Cross-lagged panel models, showing mutual influences of parental feeding practices and child weight status across 3 time points.
Notes:
Figures show paths with significant standardized regression coefficients. Only significant paths (p < .05) are shown for all models.
Covariates included parental BMI, SES, Spanish language acculturation, and child pubertal status (and child age in BMI models). Paths between covariates and other variables are not shown.
Models without significant cross-lagged paths are not shown.
2/14/2014

## Slide 2
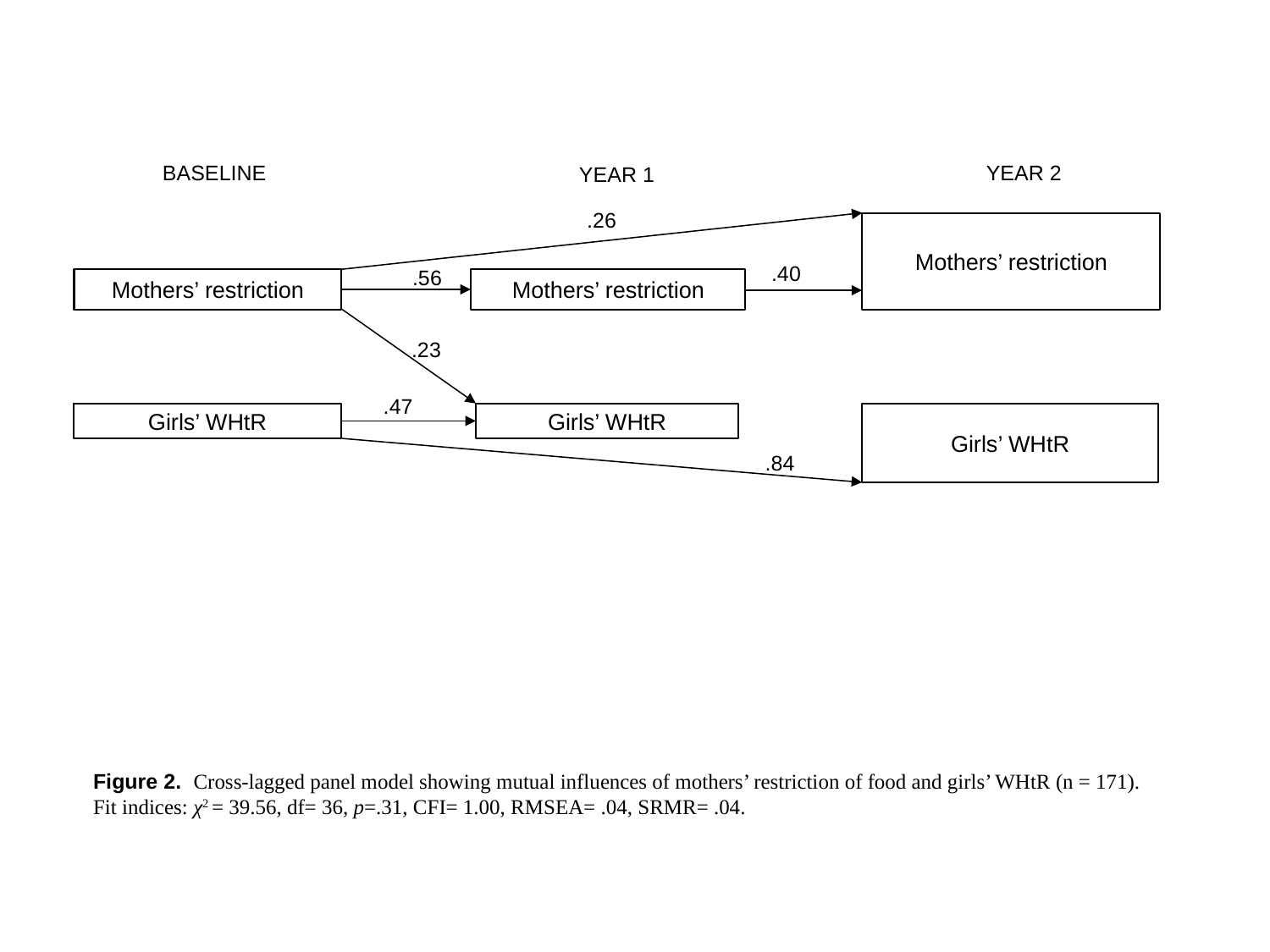

BASELINE
YEAR 2
YEAR 1
.26
Mothers’ restriction
.40
.56
Mothers’ restriction
Mothers’ restriction
 .23
.47
Girls’ WHtR
Girls’ WHtR
Girls’ WHtR
.84
Figure 2. Cross-lagged panel model showing mutual influences of mothers’ restriction of food and girls’ WHtR (n = 171).
Fit indices: χ2 = 39.56, df= 36, p=.31, CFI= 1.00, RMSEA= .04, SRMR= .04.

## Slide 3
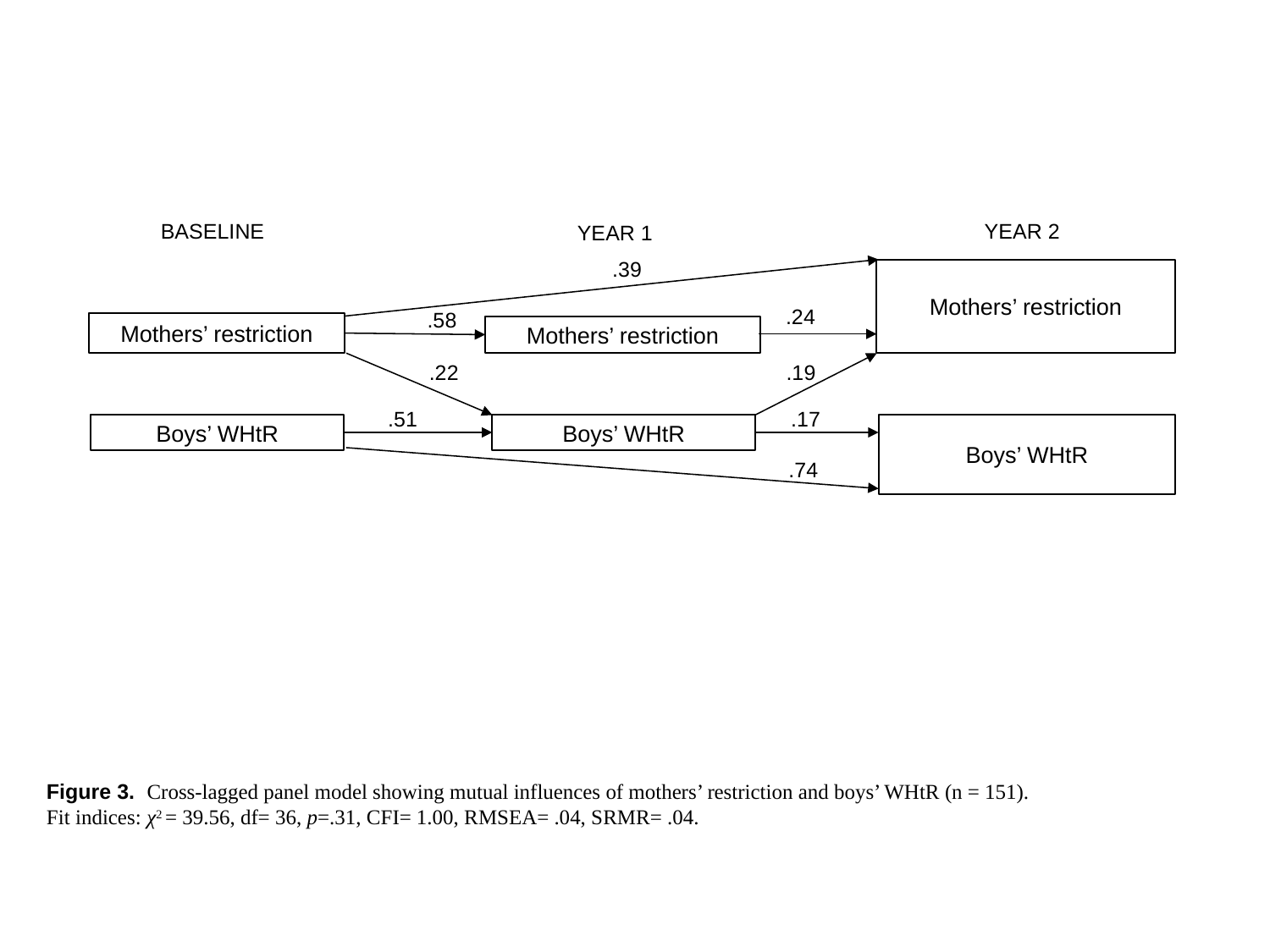

BASELINE
YEAR 2
YEAR 1
.39
Mothers’ restriction
.24
.58
Mothers’ restriction
Mothers’ restriction
 .22
 .19
.17
.51
Boys’ WHtR
Boys’ WHtR
Boys’ WHtR
.74
Figure 3. Cross-lagged panel model showing mutual influences of mothers’ restriction and boys’ WHtR (n = 151).
Fit indices: χ2 = 39.56, df= 36, p=.31, CFI= 1.00, RMSEA= .04, SRMR= .04.

## Slide 4
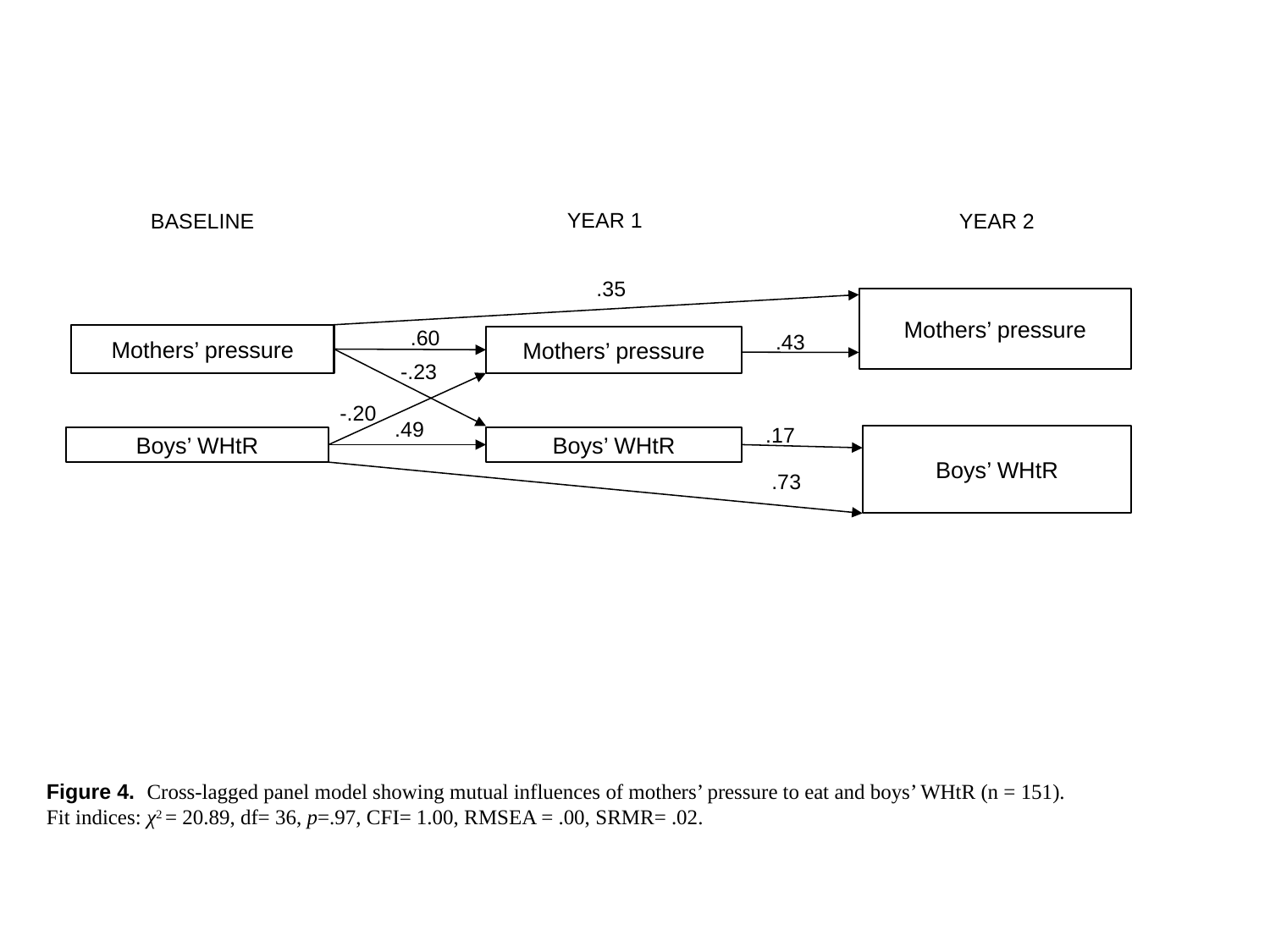

YEAR 1
BASELINE
YEAR 2
.35
Mothers’ pressure
.60
.43
Mothers’ pressure
Mothers’ pressure
 -.23
 -.20
.49
.17
Boys’ WHtR
Boys’ WHtR
Boys’ WHtR
.73
Figure 4. Cross-lagged panel model showing mutual influences of mothers’ pressure to eat and boys’ WHtR (n = 151).
Fit indices: χ2 = 20.89, df= 36, p=.97, CFI= 1.00, RMSEA = .00, SRMR= .02.

## Slide 5
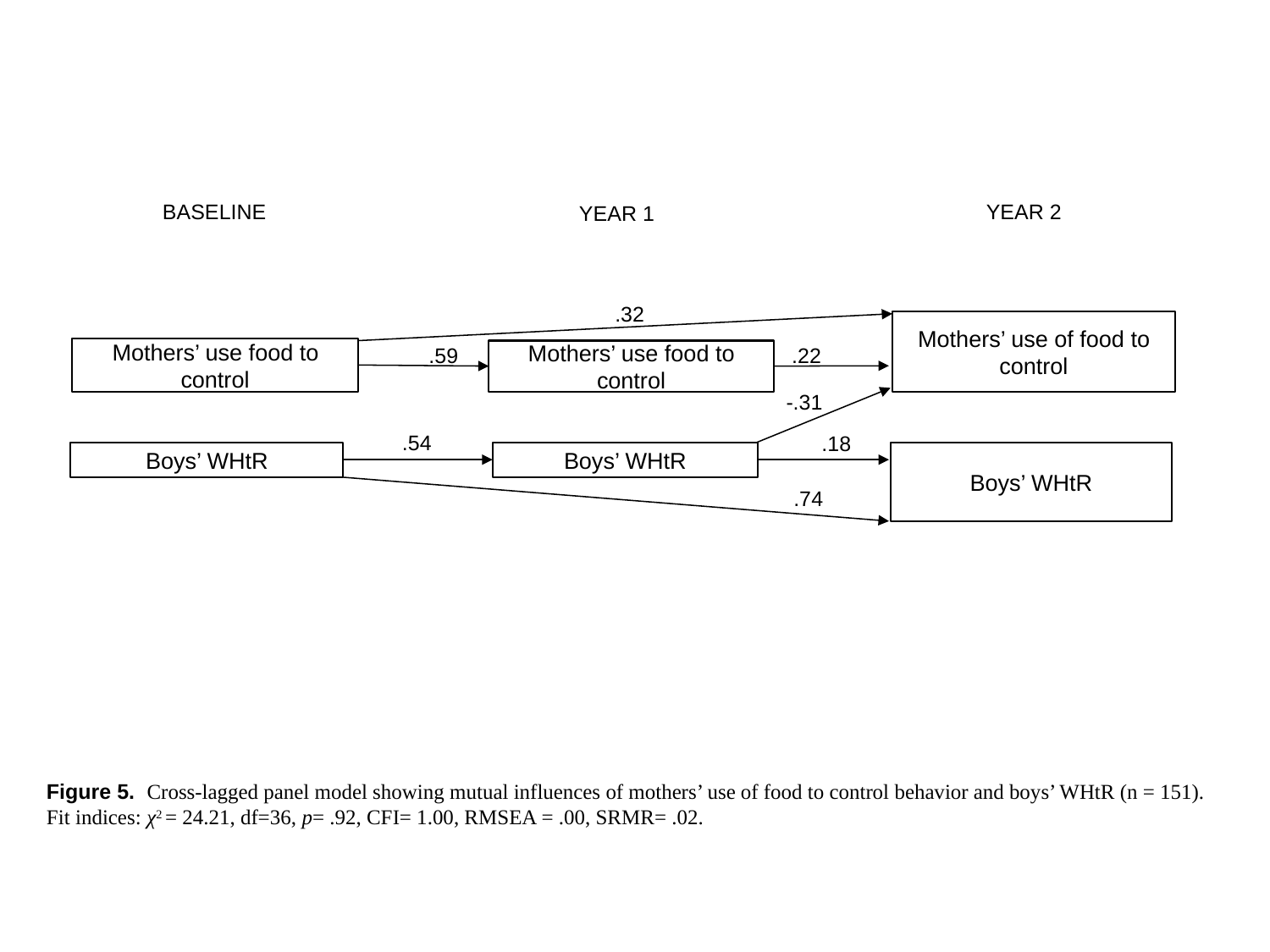

BASELINE
YEAR 2
YEAR 1
.32
Mothers’ use of food to control
.59
.22
Mothers’ use food to control
Mothers’ use food to control
-.31
.54
.18
Boys’ WHtR
Boys’ WHtR
Boys’ WHtR
.74
Figure 5. Cross-lagged panel model showing mutual influences of mothers’ use of food to control behavior and boys’ WHtR (n = 151). Fit indices: χ2 = 24.21, df=36, p= .92, CFI= 1.00, RMSEA = .00, SRMR= .02.

## Slide 6
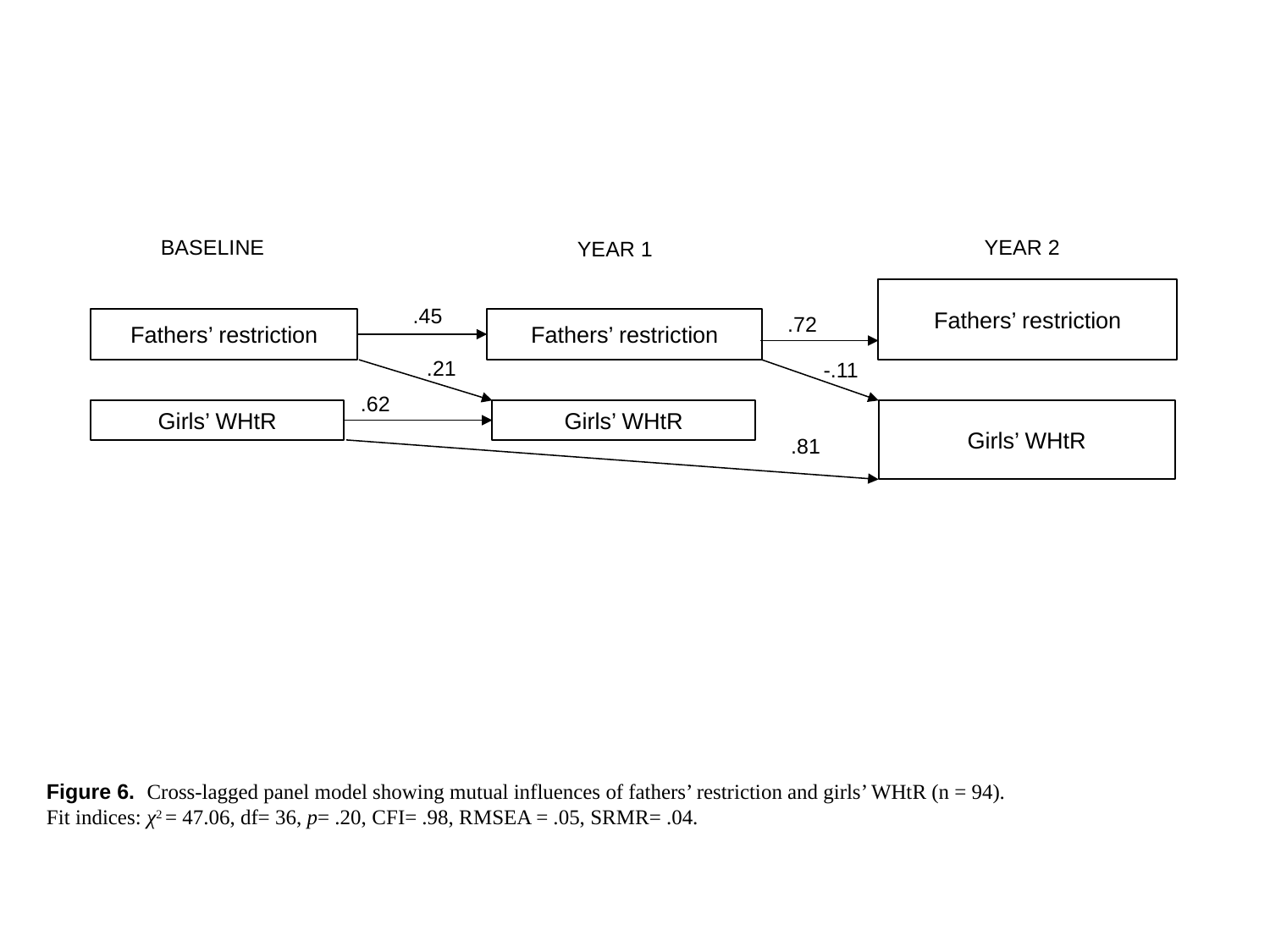

BASELINE
YEAR 2
YEAR 1
Fathers’ restriction
.45
.72
Fathers’ restriction
Fathers’ restriction
 .21
 -.11
.62
Girls’ WHtR
Girls’ WHtR
Girls’ WHtR
.81
Figure 6. Cross-lagged panel model showing mutual influences of fathers’ restriction and girls’ WHtR (n = 94).
Fit indices: χ2 = 47.06, df= 36, p= .20, CFI= .98, RMSEA = .05, SRMR= .04.

## Slide 7
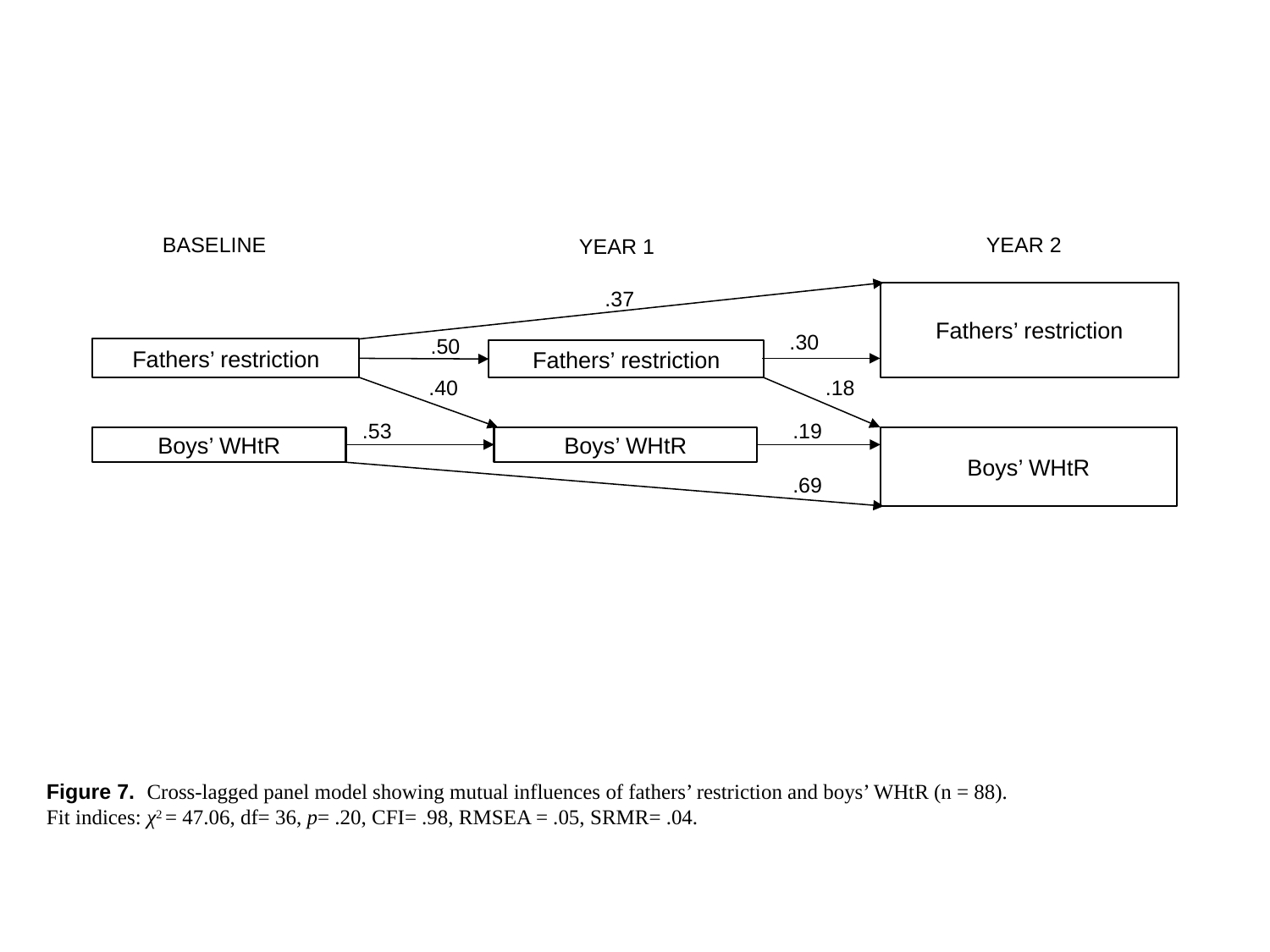

BASELINE
YEAR 2
YEAR 1
.37
Fathers’ restriction
.30
.50
Fathers’ restriction
Fathers’ restriction
 .40
 .18
.53
.19
Boys’ WHtR
Boys’ WHtR
Boys’ WHtR
.69
Figure 7. Cross-lagged panel model showing mutual influences of fathers’ restriction and boys’ WHtR (n = 88).
Fit indices: χ2 = 47.06, df= 36, p= .20, CFI= .98, RMSEA = .05, SRMR= .04.

## Slide 8
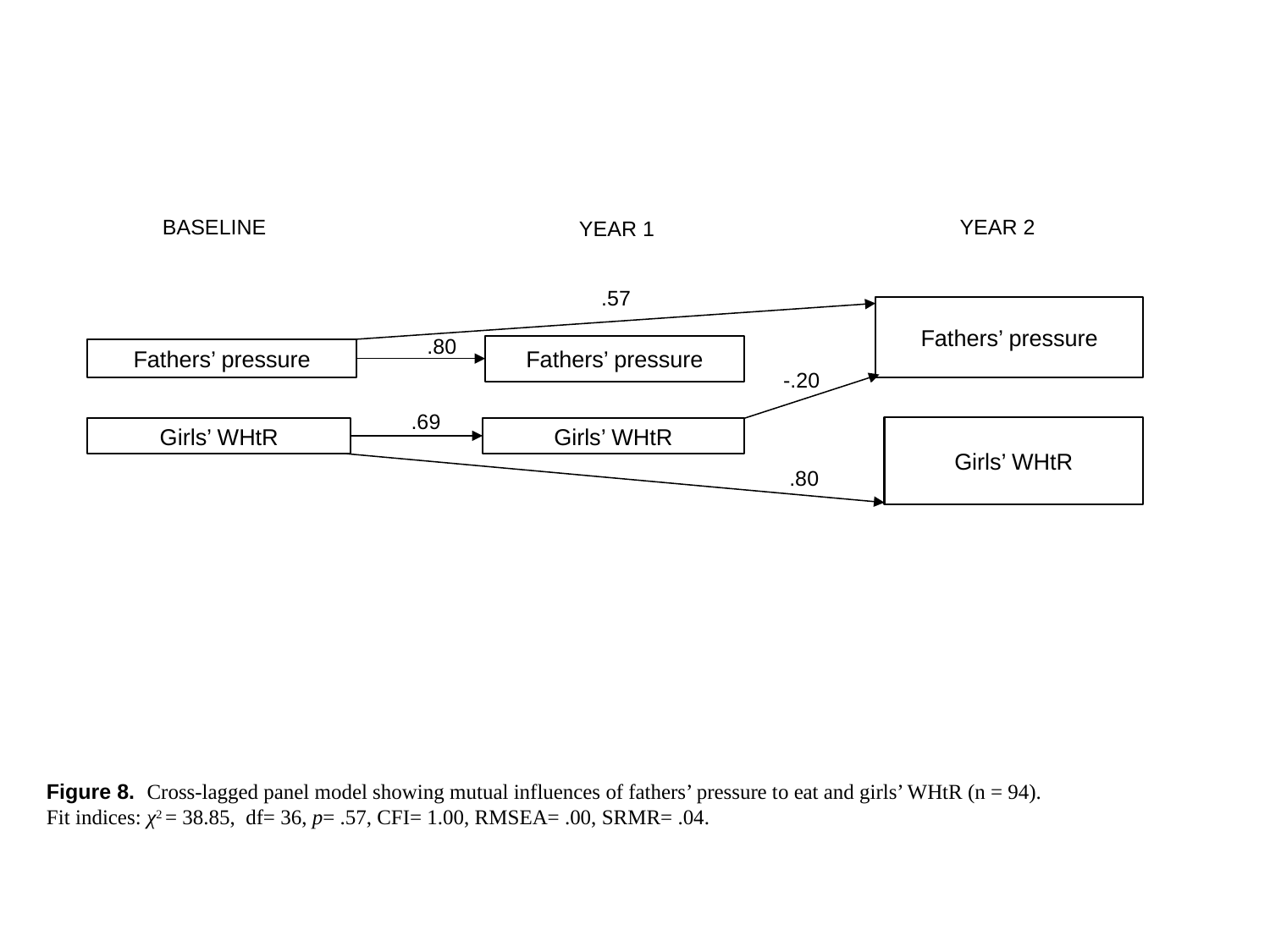

BASELINE
YEAR 2
YEAR 1
.57
Fathers’ pressure
.80
Fathers’ pressure
Fathers’ pressure
.69
Girls’ WHtR
Girls’ WHtR
Girls’ WHtR
.80
 -.20
Figure 8. Cross-lagged panel model showing mutual influences of fathers’ pressure to eat and girls’ WHtR (n = 94).
Fit indices: χ2 = 38.85, df= 36, p= .57, CFI= 1.00, RMSEA= .00, SRMR= .04.

## Slide 9
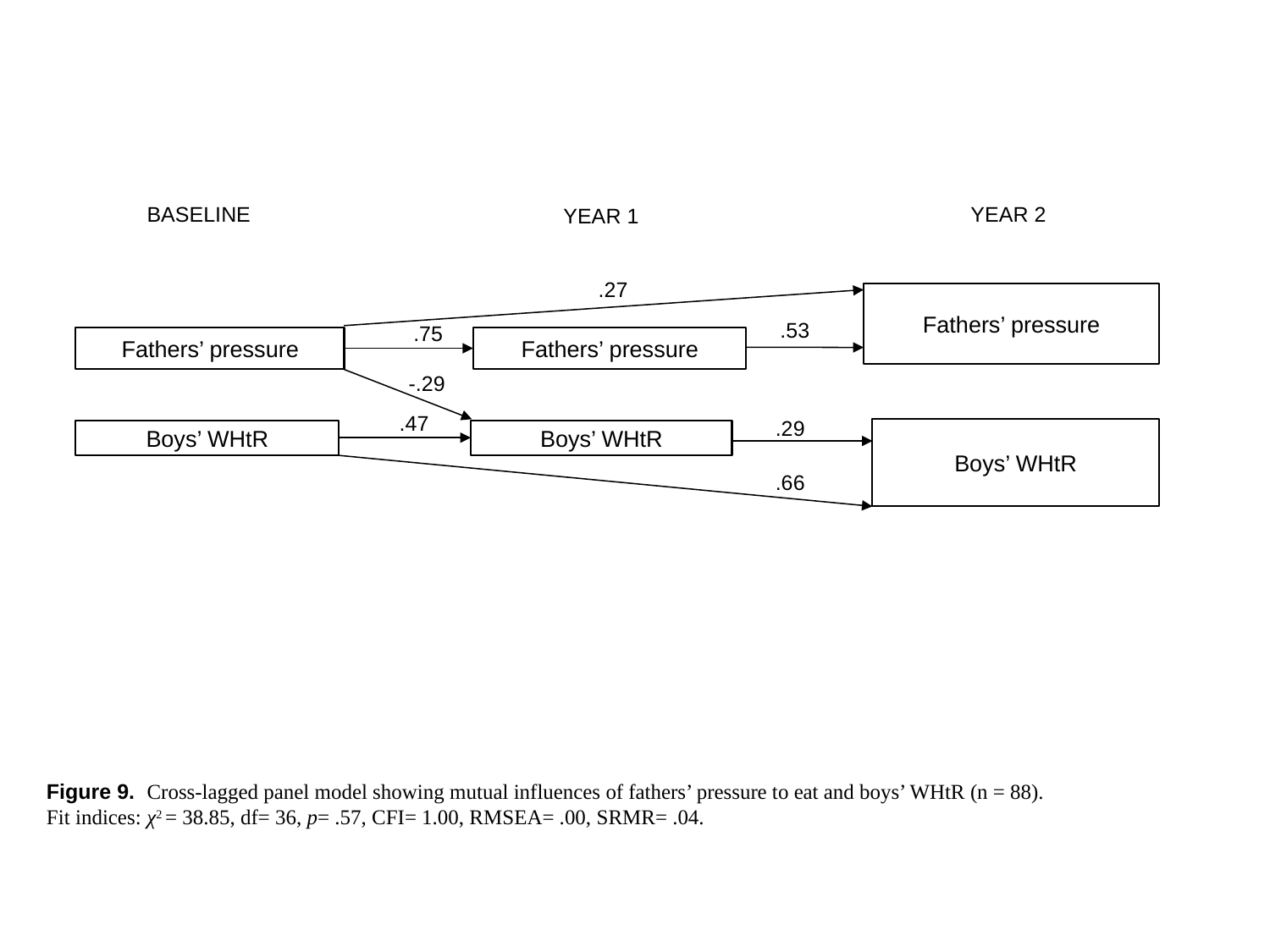

BASELINE
YEAR 2
YEAR 1
.27
Fathers’ pressure
.53
.75
Fathers’ pressure
Fathers’ pressure
 -.29
.47
.29
Boys’ WHtR
Boys’ WHtR
Boys’ WHtR
.66
Figure 9. Cross-lagged panel model showing mutual influences of fathers’ pressure to eat and boys’ WHtR (n = 88).
Fit indices: χ2 = 38.85, df= 36, p= .57, CFI= 1.00, RMSEA= .00, SRMR= .04.

## Slide 10
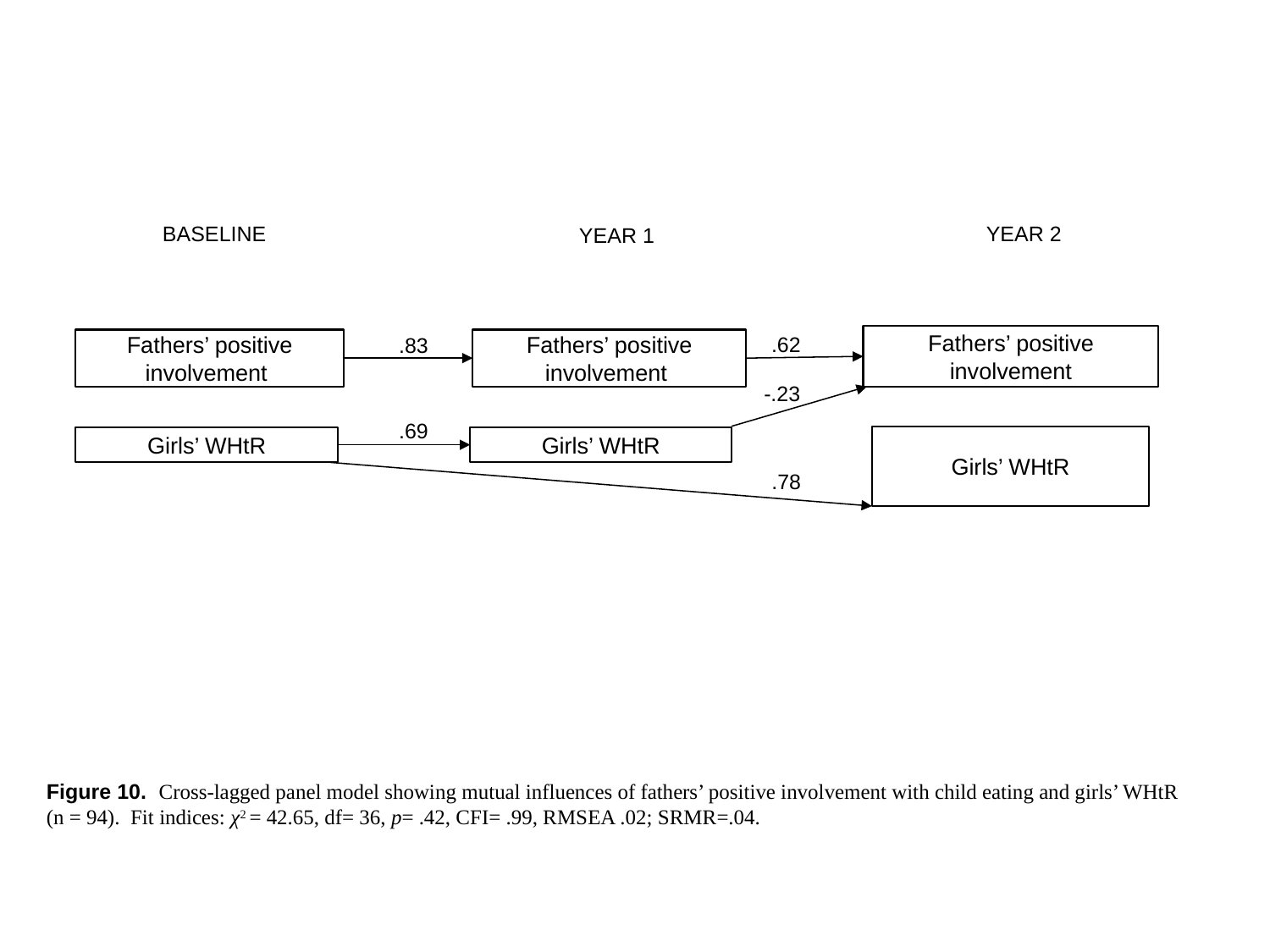

BASELINE
YEAR 2
YEAR 1
.62
.83
Fathers’ positive involvement
Fathers’ positive involvement
Fathers’ positive involvement
.69
Girls’ WHtR
Girls’ WHtR
Girls’ WHtR
.78
 -.23
Figure 10. Cross-lagged panel model showing mutual influences of fathers’ positive involvement with child eating and girls’ WHtR (n = 94). Fit indices: χ2 = 42.65, df= 36, p= .42, CFI= .99, RMSEA .02; SRMR=.04.

## Slide 11
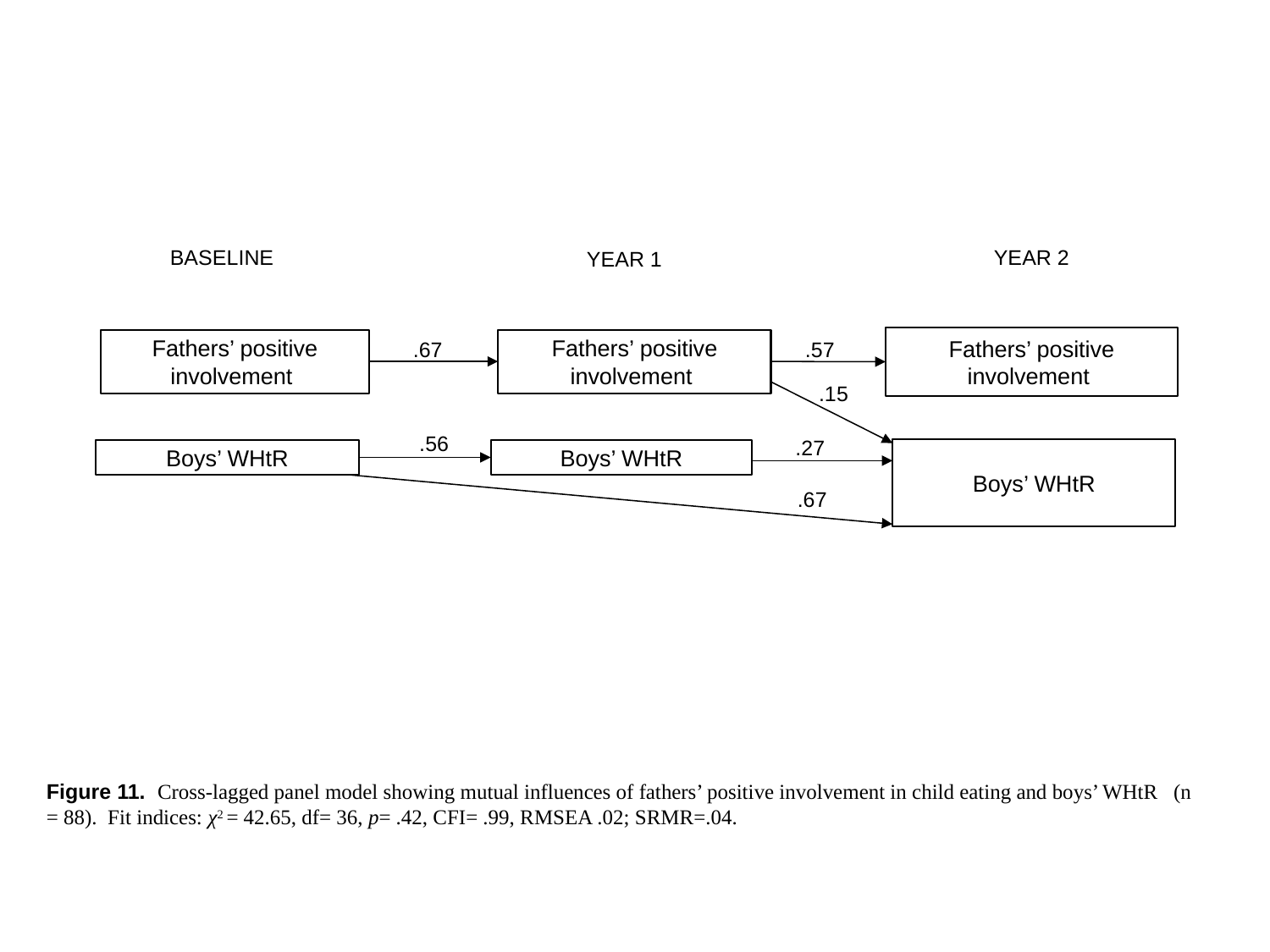

BASELINE
YEAR 2
YEAR 1
Fathers’ positive involvement
.67
.57
Fathers’ positive involvement
Fathers’ positive involvement
.15
.56
.27
Boys’ WHtR
Boys’ WHtR
Boys’ WHtR
.67
Figure 11. Cross-lagged panel model showing mutual influences of fathers’ positive involvement in child eating and boys’ WHtR (n = 88). Fit indices: χ2 = 42.65, df= 36, p= .42, CFI= .99, RMSEA .02; SRMR=.04.

## Slide 12
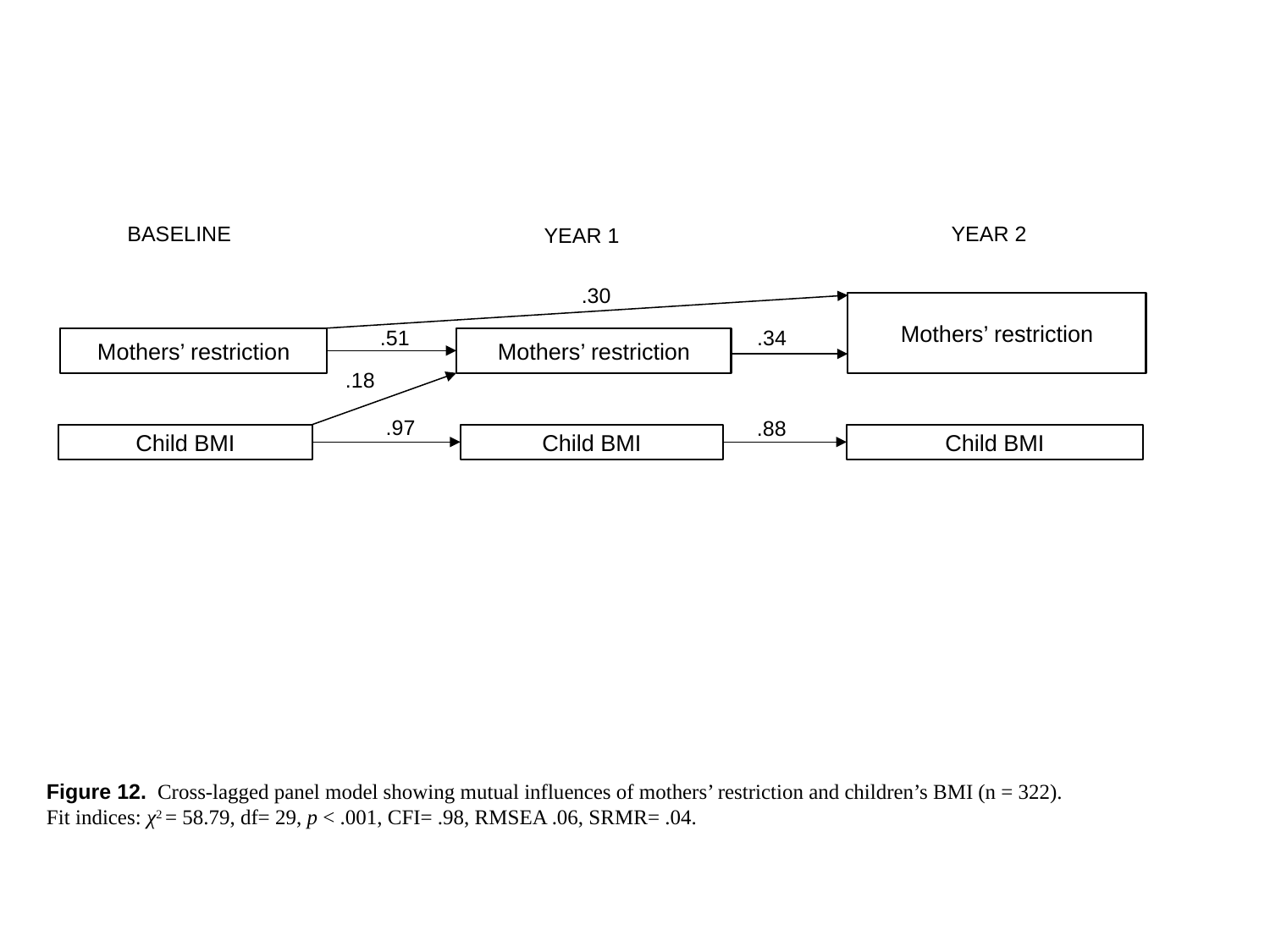

BASELINE
YEAR 2
YEAR 1
.30
Mothers’ restriction
.34
.51
Mothers’ restriction
Mothers’ restriction
 .18
.97
.88
Child BMI
Child BMI
Child BMI
Figure 12. Cross-lagged panel model showing mutual influences of mothers’ restriction and children’s BMI (n = 322).
Fit indices: χ2 = 58.79, df= 29, p < .001, CFI= .98, RMSEA .06, SRMR= .04.

## Slide 13
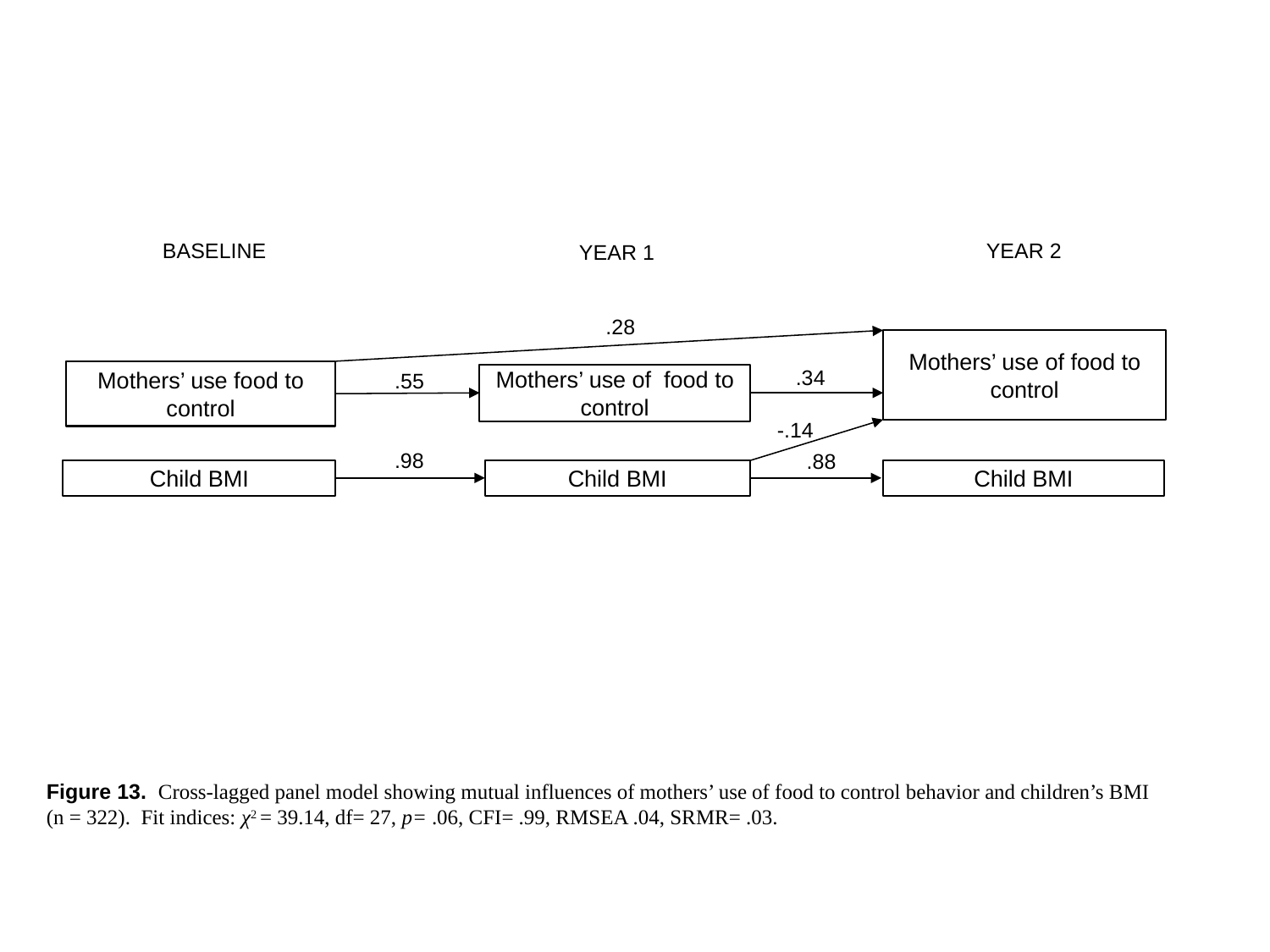

BASELINE
YEAR 2
YEAR 1
.28
Mothers’ use of food to control
.34
Mothers’ use food to control
.55
Mothers’ use of food to control
-.14
.98
.88
Child BMI
Child BMI
Child BMI
Figure 13. Cross-lagged panel model showing mutual influences of mothers’ use of food to control behavior and children’s BMI (n = 322). Fit indices: χ2 = 39.14, df= 27, p= .06, CFI= .99, RMSEA .04, SRMR= .03.

## Slide 14
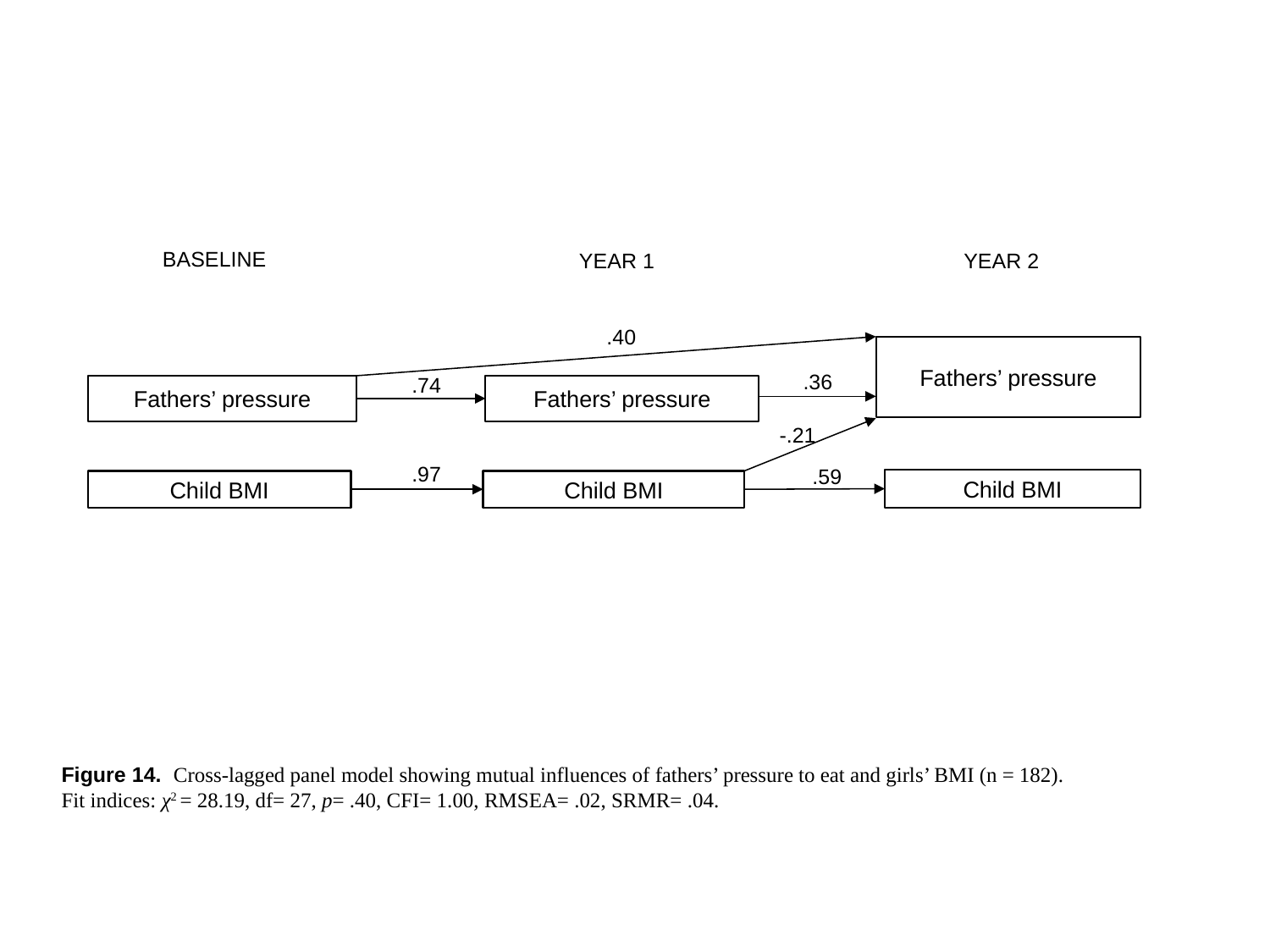

BASELINE
YEAR 2
YEAR 1
.40
Fathers’ pressure
.74
Fathers’ pressure
Fathers’ pressure
-.21
.97
Child BMI
Child BMI
Child BMI
.36
.59
Figure 14. Cross-lagged panel model showing mutual influences of fathers’ pressure to eat and girls’ BMI (n = 182).
Fit indices: χ2 = 28.19, df= 27, p= .40, CFI= 1.00, RMSEA= .02, SRMR= .04.
